# Sexual and reproductive dysfunctions induced by chronic manganese exposure: Roles of neuroaffective and olfactory impairments

**DOI:** 10.1192/j.eurpsy.2025.2307

**Published:** 2025-08-26

**Authors:** H. Harifi, H. Hami, L. Bikjdaouene

**Affiliations:** Laboratory of Biology and Health, Faculty of Science, Ibn Tofail University, Kenitra, Morocco

## Abstract

**Introduction:**

Reproduction in mammals relies on complex interactions involving the genital and olfactory systems, which can be influenced by environmental factors, such as manganese (Mn). Although essential for survival, Mn is potentially toxic over long periods, potentially affecting sexual and reproductive behaviors.

**Objectives:**

This study aims to assess the long-term effects of Mn exposure on sexual and reproductive functions in male Wistar rats, focusing on Mn-induced neuroaffective and olfactory dysfunctions.

**Methods:**

Male Wistar rats received intraperitoneal injections of Mn at doses of 6 mg/kg, 25 mg/kg, and 30 mg/kg for 12 weeks. Each experimental group consisted of one Mn-intoxicated male and four non-intoxicated females. After six days of cohabitation, the females were isolated to evaluate fertility outcomes. The study also monitored weight changes and conducted behavioral assessments for anxiety, depression, and olfactory functions in males.

**Results:**

Higher Mn doses (25 mg/kg and 30 mg/kg) resulted in significant behavioral changes in males, including anxiety, depression, and olfactory dysfunctions, which were associated with decreased reproductive success. Specifically, pregnancy rates were 33% (4 out of 12) at 25 mg/kg and zero at 30 mg/kg. In contrast, males treated with 6 mg/kg Mn exhibited no significant neuroaffective or olfactory impairments, maintaining fertility rates comparable to those of the control groups.

**Conclusions:**

Chronic Mn exposure adversely affects sexual behavior and reproductive success in male Wistar rats, probably due to olfactory and neuroaffective disruptions. Further research is recommended to elucidate the mechanisms underlying these effects.

**Disclosure of Interest:**

None Declared

